# Acceptability, feasibility and utility of a Mobile health family planning decision aid for postpartum women in Kenya

**DOI:** 10.1186/s12978-019-0767-9

**Published:** 2019-07-08

**Authors:** Rubee Dev, Nancy F. Woods, Jennifer A. Unger, John Kinuthia, Daniel Matemo, Shiza Farid, Emily R. Begnel, Pamela Kohler, Alison L. Drake

**Affiliations:** 1 Dhulikhel Hospital, Kathmandu University Hospital, Kavre, Nepal; 2 Department of Biobehavioral Nursing and Health Informatics, University of Washington, Seattle, WA USA; 3 Department of Obstetrics and Gynecology, University of Washington, Seattle, WA USA; 4 Department of Research and Programs, Kenyatta National Hospital, Nairobi, Kenya; 5 Department of Global Health, University of Washington, Seattle, WA USA; 6 Department of Psychosocial and Community Health & Department of Global Health, University of Washington, Seattle, WA USA

**Keywords:** Postpartum, Family planning, Contraceptive counseling, Decision aid

## Abstract

**Background:**

Unmet need for contraception is high during the postpartum period, increasing the risk of unintended subsequent pregnancy. We developed a client facing mobile phone-based family planning (FP) decision aid and assessed acceptability, feasibility, and utility of the tool among health care providers and postpartum women.

**Methods:**

Semi-structured in-depth interviews (IDIs) were conducted among postpartum women (*n* = 25) and FP providers (*n* = 17) at 4 Kenyan maternal and child health clinics, 2 in the Nyanza region (Kisumu and Siaya Counties) and 2 in Nairobi. Stratified purposive sampling was used to enroll postpartum women and FP providers. Data were analyzed using an inductive content analysis approach by 3 independent coders, with consensual validation.

**Results:**

FP providers stated that the Interactive Mobile Application for Contraceptive Choice (iMACC) tool contained the necessary information about contraceptive methods for postpartum women and believed that it would be a useful tool to help women make informed, voluntary decisions. Most women valued the decision aid content, and described it as being useful in helping to dispel myths and misconceptions, setting realistic expectations about potential side effects and maintaining confidentiality. Both women and providers expressed concerns about literacy and lack of familiarity with smart phones or tablets and suggested inclusion of interactive multimedia such as audio or videos to optimize the effectiveness of the tool.

**Conclusions:**

The iMACC decision aid was perceived to be an acceptable tool to deliver client-centered FP counseling by both women and providers. Counseling tools that can support FP providers to help postpartum women make informed and individualized FP decisions in resource-limited settings may help improve FP counseling and contraceptive use in the postpartum period.

## Plain English summary

We explored feasibility, acceptability and utility of an Interactive Mobile Application for Contraceptive Choice (iMACC) client focused decision aid designed to support family planning (FP) counseling and uptake. This qualitative study was conducted at 4 Kenyan maternal and child health clinics: 2 rural sites in the Nyanza region and 2 urban sites in Nairobi. We recruited 25 postpartum adolescents’ girls and women (age ≥ 14 years) and 17 FP providers (nurses) for in-depth interviews. Overall, women and providers felt that the decision aid was easy to use and had all the necessary information on different contraceptive methods that would help them in decision making. They further reported that such a decision aid will help them get rid of myths and misconceptions associated with the contraceptive methods and will also keep their information confidential. Both women and providers expressed concerns about literacy and technological challenges of using smart phones or devices and suggested inclusion of multimedia such as audio or videos to optimize the effectiveness of the tool. Overall, the iMACC decision aid was considered as a new and informative FP tool that has potential to provide need specific contraceptive counseling to postpartum women.

## Background

Addressing unmet need for family planning (FP) to prevent unintended pregnancies is a high priority for women’s health, and is an effective strategy to reduce both maternal and infant morbidity and mortality [1]. Over 200 million women and girls in low- and middle-income countries (LMICs) who desire to space or limit pregnancy are not using modern contraception, and may lack access to FP information, FP services, and effective methods of FP [2, 3]. Improving contraceptive counseling efforts may be one strategy to prevent adverse pregnancy outcomes and unsafe abortion among young women, reduce risks of maternal mortality, and promote achieving reproductive rights.

Efforts focused on reducing unmet need for contraception and unintended pregnancy rates among postpartum adolescents and young women, specifically, may be strategic. The postpartum period provides a unique opportunity to address women’s contraceptive needs and provide high quality information on contraceptive options while women are engaged in care. While 91% of postpartum women in LMICs report a desire to delay subsequent pregnancies for at least a year [4], women are often discharged from health care facilities without a plan to initiate contraception or a contraceptive method to use. As a result, over 60% of women in these settings have unmet need for FP during the 2-year postpartum period [4, 5]. Among women who use postpartum FP in LMICs, more than half rely on short-acting, user-dependent methods [5]. In Kenya 59% of women reported using modern contraception by 9 months postpartum, while only 12% used long-acting reversible contraception (LARC) [6].

Individual, provider, and societal barriers to FP contribute to sub-optimal modern contraceptive prevalence rates (mCPR) and limited LARC use among postpartum women in Kenya [7]. Key barriers to postpartum contraceptive use include insufficient knowledge of suitable FP methods, fear of side effects, and myths and misconceptions about contraception [8, 9]. In addition, lack of provider time and opportunities to offer FP counseling may contribute to low contraceptive use during the postpartum period in busy MCH clinics [10]. FP counseling could address knowledge gaps; however, it is often not delivered during the postpartum period or tailored to individual needs, making it difficult for women to retain key messages and make informed decisions [11]. Both postpartum women and providers have articulated the need for augmented services to support quality contraceptive counseling [8]. Thus, novel approaches to deliver comprehensive, client-centered FP counseling that address individual and structural barriers are essential to increase mCPR among postpartum women and improve contraceptive method-mix.

The rapid expansion of mobile devices in resource-limited settings has led to development of tools that remotely deliver health information and allow for communication with patients [12]. While SMS interventions have been shown to be effective in efforts to improve health outcomes for chronic diseases, treatment adherence, exclusive breastfeeding and early postpartum contraception in LMICs [13–18], rigorous evaluations of mobile health (mHealth) technology to support healthcare decision-making have been limited [15, 16]. Decision support tools have been effective in helping women select contraceptive methods and improve method use in the United States, but have not been studied in resource-limited settings [19]. The World Health Organization (WHO) introduced a postpartum family planning (PPFP) compendium to address the needs of postpartum women, but it is limited to the English language and mainly focuses on medical-eligibility criteria (MEC) [20]. Client facing mHealth contraceptive decision aids that could be incorporated into existing FP clinics to help women make informed contraceptive decisions in resource-limited settings have not been evaluated.

We developed a FP decision aid (*Interactive Mobile Application for Contraceptive Choice* [iMACC]) designed to help prepare postpartum women to make personalized deliberate contraceptive choices and streamline FP counseling in Kenya. In this study, we interviewed postpartum women and FP providers to evaluate the acceptability and feasibility of the iMACC mobile decision aid in Kenya.

## Methods

### Study design

We conducted a cross-sectional qualitative study to evaluate the acceptability and feasibility of the self-administered iMACC decision aid. While the decision aid was designed for use by postpartum women, we also included providers to assess perceptions of utility to complement counseling offered by providers, or to be used with providers for women who could not use it on their own. Study participants were also asked to provide feedback for future refinement of the decision aid. Semi-structured in-depth individual interviews (IDIs) were conducted among postpartum women and FP providers/nurses in Kenya.

### Study setting

The study was conducted at 4 Kenyan maternal child health (MCH) clinics: 2 rural sites in the Nyanza region (Kisumu and Siaya Counties) and 2 urban sites in Nairobi. All MCH clinics are high-volume government-run public health facilities that serve low-income populations. The study sites were co-located within these facilities, but do not provide any clinical care.

### Study recruitment and sampling

Study staff recruited postpartum adolescent girls (age 14–21 years) and young adult women (age 22–24) attending 6-week infant immunization clinic visits using stratified purposive sampling method. Women waiting for the immunization services who could read in either English, Kiswahili, or Dholuo were approached by study staff and invited to participate in the study. We also recruited FP providers by using simple purposive sampling method; all FP providers were nurses. FP providers were referred to study staff by clinic administrators (i.e., matron). The sample size was guided by achieving thematic saturation, when no new data or themes emerged from interviews [21]. A range of 20–30 interviews has previously been reported to be adequate for sampling among a homogenous population [22].

### Ethical considerations

This study was approved by the Institutional Review Board of the University of Washington (STUDY00001916) and the Kenyatta National Hospital-University of Nairobi Ethics and Research Committee (P252/05/2017). All participants provided written informed consent. Adolescents ≥14 years who have previously been pregnant are considered emancipated minors in Kenya and were able to provide their own written consent [23].

### Description of Mobile app

**Fig. 1:**
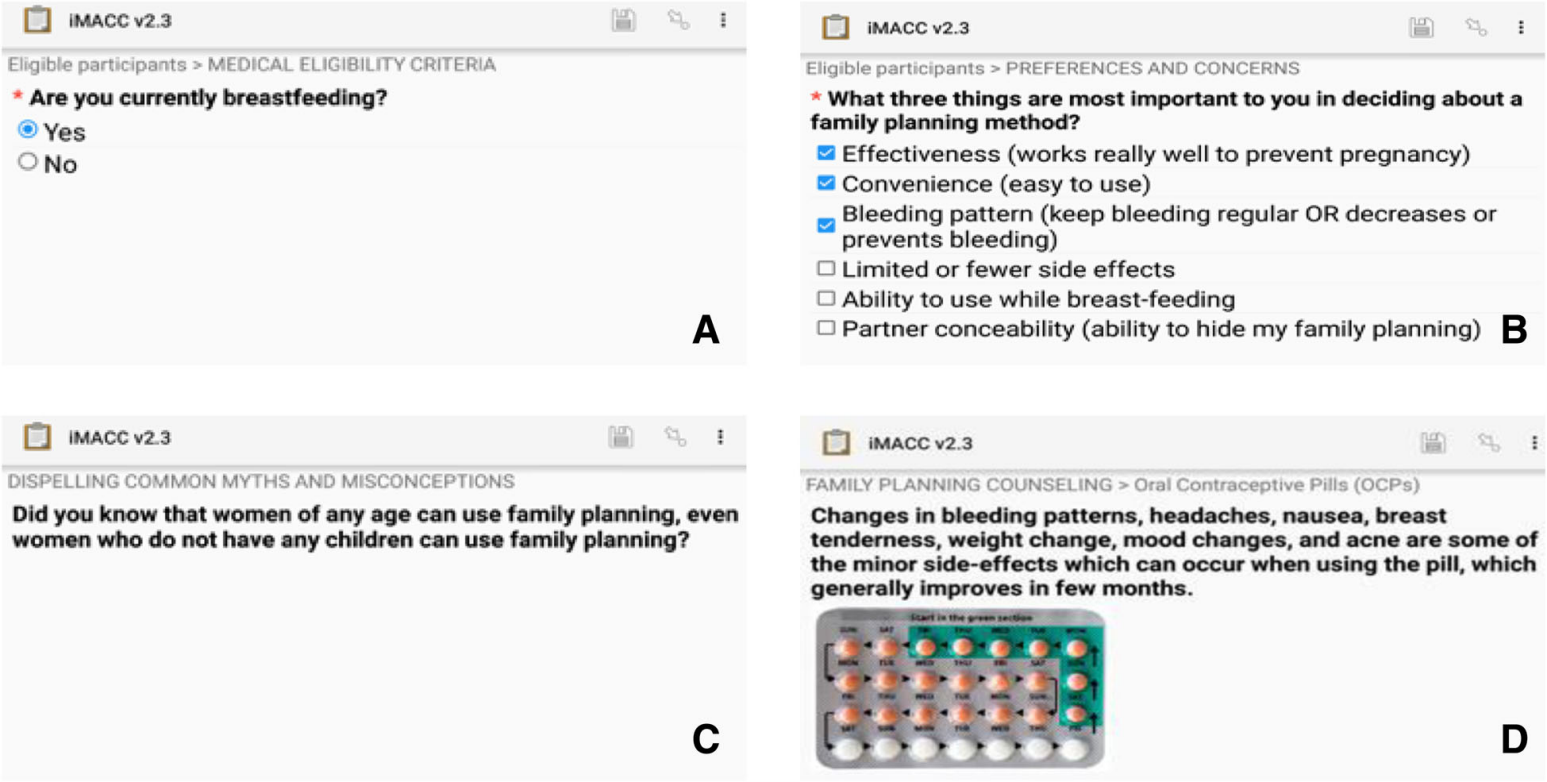
Interactive mobile application for contraceptive choice (iMACC) screen shots of selected content of (**a**) Assessment of medical eligibility criteria (**b**) Assessment of preferences and concerns (**c**) Dispelling common myths and misconceptions (**d**) Provision of information about oral contraceptive pills

iMACC is a client-facing mobile application designed to provide systematic, yet personalized, contraceptive counseling to postpartum women, guide postpartum women through FP decision-making, and provide accurate information about FP methods, including method characteristics and potential side effects. Based on the WHO classification of digital health interventions, clients or postpartum women are the targeted primary user of the application [24]. iMACC was developed for self-administration for women to use while waiting to see a FP provider at the facility; however, if women were unable to use it on their own they could elect to have study staff assist. The application was developed using Open Data Kit (ODK), an open source Android™-based application that renders standard forms (XLS form) on a tablet. The prototype was designed by study team members and nurses in Kenya, and allows women to select one of three languages: English, Kiswahili, or Dholuo (Fig. 1).

iMACC combines images and text in a heuristic approach to provide tailored contraceptive counseling to women. It features 14 health history inquiries to assess some of the most common exclusions for FP based on the WHO MEC [25], followed by 48 statements and/or inquiries to assess individual experiences, preferences and concerns about FP. To assess FP preferences, women are asked questions about fertility intentions and return to fertility, convenience of use and concealability, partner support for FP, perceptions about potential changes in bleeding patterns due to contraceptive use, side effect concerns, cost of FP, and frequency of administration/dosing. Following the assessment, women are provided counseling on 6 modern methods (oral contraceptive pills, injectables, implants, intra-uterine devices, male condoms, and male and female sterilizations) through the tool.

### Theoretical framework

**Fig. 2:**
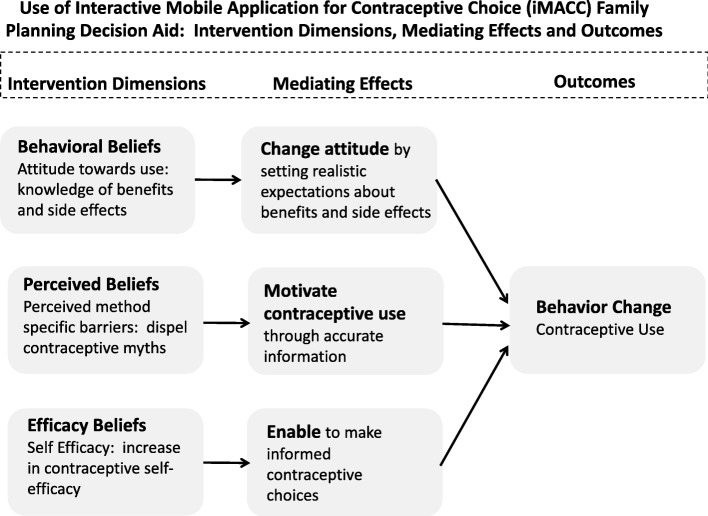
Integrated theoretical framework guiding contraceptive use

iMACC content was guided by 3 behavioral theories, the Theory of Planned Behavior (TPB) [26], the Health Belief Model (HBM) [27], and Social Cognitive Theory (SCT) [28], which encompass elements of behavioral, perceived, and efficacy beliefs. Each of these theories have been successfully used to impact contraceptive behavior in resource-limited settings [29–32]. In this framework, behavioral beliefs from TPB are hypothesized to change the attitude of women towards contraceptive use by providing realistic expectations of benefits and side effects of contraceptive methods. Perceived beliefs from HBM are hypothesized to motivate contraceptive use by providing accurate method specific information. Efficacy beliefs from SCT are hypothesized to empower women to make informed decision-making. Overall, the integrated theoretical framework for iMACC (Fig. 2) suggests the tool could impact contraceptive behavior by affecting these 3 sets of beliefs, leading to contraceptive initiation and continuation.

### Study procedures

All study participants completed iMACC independently after instructions were provided by study staff. Following completion of the application module of iMACC, women and FP providers were individually interviewed by 1 of 2 social scientists experienced in conducting qualitative interviews. IDIs were audio recorded using a digital recorder and transcribed and translated after IDI completion. Feasibility was assessed with question about ease of use; ability to understand content, clarity and adequacy of information provided; appropriateness of length of time required to complete iMACC; and need for clarification and/or semantics [33]. Acceptability was assessed through inquiries related to the tool features, technological acceptability (relative advantage over other available decision aids in the clinic e.g., flip charts, intention to use), preferences over the content, and content satisfaction [34–36]. Utility was assessed by inquiring about perception of usefulness of the tool in making informed decisions, reported improvements in knowledge on range of contraceptive options and health concerns, and self-efficacy in readiness for contraceptive uptake [37, 38].

### Data analysis

Transcripts were analyzed using qualitative content analysis, which organizes informational content of textual data into categories derived from the data [39]. A team of 3 coders (RD, SF, ERB) created a comprehensive codebook containing 84 code descriptions. The 3 coders independently coded 33% (*n* = 14) of the transcripts and compared their coding to identify any inconsistencies. The primary coder (RD) reviewed the coding of all transcripts. Disagreements on thematic codes were resolved through discussion, thus establishing inter-coder agreement and performing investigator triangulation [40]. During the coding process, major themes were identified, organized into concepts, and relationships between concepts were examined. The thematic framework was refined throughout the analytic process by identifying new themes and expanding existing ones. As codes were classified into larger themes, we also searched for meaningful patterns by age of postpartum women (adolescents and young adults) using the constant comparison method. However, since we did not detect significant differences in themes between age groups or clinics, we combined perspectives of all postpartum women in our results.

To enhance robustness of results, a consensus on the final results was made with all 3 coders. Dedoose (Los Angeles, California, Version 8.1) was used to maintain an electronic database of transcripts and organize coded data.

## Results

A total of 25 postpartum women and 17 FP providers were enrolled. Twelve (48%) postpartum women were from rural MCH clinics, and 13 (52%) from urban clinics; 15 (60%) were adolescents and young women between the ages of 14–21 (Table 1). FP providers were between 18 and 58 years of age.

**Table 1:** Descriptive characteristics of postpartum women (*N* = 25)

Characteristics	N	n (%)
Age (14–21 years)	25	15 (60%)
Currently married	25	19 (76%)
Secondary education or below	25	20 (80%)
Parity of women	25	
1		10 (40%)
2–3		12 (48%)
> 3		3 (12%)
MCH centers	25	
Rural		12 (48%)
Urban		13 (52%)

FP (family planning); MCH (maternal and child health)

### Perceived feasibility

#### Organization, ease of use, and comprehension

Nearly all women and providers (98%) felt the FP decision aid application was self-explanatory and easy to use. Women noted the ease of navigation through the tool, stating *“all I need to do is answer the questions and swipe.”* Overall, both women and providers thought content was presented in a systematic and simple manner. All providers reported the flow of the material to be logical and easy for women to understand. Some women even commented that they could anticipate upcoming inquiries: “*when you are navigating you can even guess the next question because the way the questions have been arranged they have a flow, it’s not jumping on any question, but they have a very systematic flow*.” The majority of women (92%) reported no challenges in answering questions or comprehending iMACC content. Both women and providers felt that the content reflected critical information often requested during FP counseling and included key components necessary to make contraceptive decisions. One provider said:

*“I feel the information is very important because it has picked on the specific things that clients normally ask about family planning methods. So, to me it’s important and it’s also detailed.” – FP provider, 29 years*

Overall, the language was appropriate for women to use on their own. However, both providers and women noted some medical terms (e.g., aura, blood clot, hypertension, and conception) they believed would be difficult for lay audiences to understand. For example, one provider said:

*“…for that word aura and blot clotting in the lung and leg it will be a bit challenging. Some of them don’t even know where the lung is and then they will be wondering blood clot and might think it is a big thing.” – FP provider, 20 years*

#### Time required, use of technology, and literacy

Participants completed iMACC in a mean of 15 min. All women said the length of the time was *“just sufficient, not too much or too little.”* However, several providers (*n* = 3) disagreed expressing concerns about the amount of time it would take to complete. They felt the duration would be dependent on how fast someone is able to read and comprehend. Based on experience providing FP counseling, one provider said:

“*For somebody new who has never known about FP, and it is their first time they would take a longer time*.” – *FP provider, 20 years*

Several providers (*n* = 14) thought the decision aid would streamline the counseling process by reducing time to provide comprehensive counseling since they felt the decision aid included all of the information and answers to common questions asked by FP clients. They said, *“It will save time a lot because once you have given the tool to the client she has everything, so when she comes [to us] it will be just review and it will take shorter time.”* However, two providers were concerned the decision aid would increase their workload by taking a longer time to counsel women. They believed women might have more questions about a larger number of contraceptive methods after using iMACC, resulting in more time to counsel women on these additional methods.

Overall, women and providers felt the mobile design of iMACC would be an appealing feature for a decision aid, and women would be able to use it on their own; however, this opinion was not universal. In rural clinics, some providers thought the use of technology was an appealing feature of the decision aid, particularly for younger FP clients. They thought that women who knew how to use smart devices would be happy to use such an aid.

*“You know young people nowadays like smart phones, so if I can have the tool in the tablet to help educate them, they will even bring more women their peers to the clinic, because they will be saying to them, if you go there, there are tablets that tell you about family planning. It will be exciting for them. That is the generation now, it is digital” – FP provider, 18 years*

Other providers in rural clinics expressed concern over self-administration:

*“This is a rural area, there are people who have never seen or used touch screen phones, if you want them to go through that tool on their own [it] might not be very possible.”*- *FP provider, 18 years*

However, some providers and women believed there were strategies that could be used to overcome these barriers:

*“Some [women] may read [iMACC] and not understand, especially if they are not literate, so for such women someone has to guide them.”* – *Female participant, 17 years*

Both women and providers expressed concerns about literacy being a barrier to using the decision aid. Providers said they have observed that women can speak well but are not able to read or, may not able to understand the information if they are literate. They believed that for these women, using iMACC would be challenging.

*“We will have problems with clients where the education level is zero or little.”* – *FP provider, 40 years*

Women echoed similar concerns:

*“I think it is a very interesting tool and it is quite informative, but it would only work with a woman who is literate. It would not work with a woman who is semi-illiterate. Yeah, so for a woman who is literate and has the knowledge of using a smart phone, then I think that it is something that is going to be very interesting and very informative” – Female participant, 16 years*

### Perceived acceptability

#### Confidentiality supports participant engagement

iMACC was perceived to be a confidential decision aid, and this feature was recognized as a benefit by both women and providers. Women reported that there are questions adolescents do not feel comfortable asking health care providers or being asked, so having such a tool would allow them to learn information on their own. Furthermore, they said they would be more likely to answer questions honestly because they know no one would be able to identify them, as they do not have to enter their name.

*“…you know it will be between the client and the tool, nobody else will see what they are filling [answer to the questions], so it has some level of confidentiality.” – Female participant, 20 years*

Women also described the desire of adolescents to seek FP services discretely. If adolescents receive information through a tablet, others would assume they are using their phone and would not know they are receiving FP services, making the tool more acceptable. Additionally, they reported that adolescents would be able to use iMACC freely, without pressure to answer quickly or fear that someone might overhear.

“*You know, sometimes you may be asked questions, like if you asked me some question directly I may not be able to answer you, but when I am alone with the tablet I can answer.*” *– Female participant, 18 years*

#### Recommended features to improve interaction and need for additional information

While iMACC included graphics and photos to promote counseling, women and providers recommended incorporating more pictorial content and additional multimedia features (such as audio or video elements), to improve quality and promote comprehension. They felt these additional elements could support counseling and help overcome literacy barriers. One provider described what she would like to see in the tool:

*“I feel, if we could have videos to show them, it could be better, it could improve our counseling because as you are talking to the mother, maybe she can see on the screen.” – FP provider, 55 years*

Women and providers thought iMACC could include more educational content on procedures associated with LARC and permanent methods. Providers felt that women who had no experience with these methods would need more basic details about procedures to make an informed choice about LARC and permanent methods. One woman in our study echoed this sentiment:

*“I feel the information is just okay but for someone who have never used implant sometimes for example apart from reading it is important that someone explains the process further. You know people fear operations.” – Female participant, 28 years*

Some providers also thought information about female condoms should be incorporated into the decision aid. Providers said some women, or couples, preferred female over male condoms. However, women in our study did not mention the need to include information on female condoms and felt the information about male condoms did not add anything new since they already knew a lot about male condoms:

*“About condom and how to use it, that is not very important. Everybody knows about it [condoms].” – Female participant, 21 years*

While iMACC included information on contraceptive effectiveness while breastfeeding, one provider suggested also including messages on the timing during the postpartum period when it is safe to initiate specific methods:

*“I saw it is written that Depo is effective in breastfeeding but now it is always effective after 6 weeks it was not clear that way. So, it can cause confusion because some women start using FP as early as even 3 weeks after delivery.” – FP provider, 20 years*

### Perceived usefulness

#### Improved knowledge with a personalized approach

Overall, women said the decision aid would be useful for them. They reported a more thorough understanding of their contraceptive options after using the decision aid and felt it would help them choose the best option for them (Table 2). A woman concerned about safety of contraceptives during breastfeeding said, *“I felt Depo was okay because even if you are breastfeeding, it is safe.”* All women and providers noted that inclusion of information on side effects would help women gain a more comprehensive understanding of potential side effects, which was expected to improve their ability to make informed choices about contraception. For example, one woman demonstrated her understanding by saying, *“I realized that the periods, when they start skipping I should not be worried when I am using family planning and that some of it [is] normal.”*

**Table 2:** Selected quotations from the interviews of postpartum adolescents and adult women supporting the constructs of the integrated theoretical framework

Increase in knowledge	
“I have never used family planning, I always just hear about it, so I learnt today. I have learnt that there are pills that you can use, the injection, you can also do tubal ligation.” - 19 years “I have learnt about the methods of family planning that can be used during breastfeeding and what I have learnt again especially that one for three months injection you can use even if you have never delivered.” – 17 years	
Enhance realistic expectations of side effects	
“I can now decide to choose this [injectables] or the other [IUDs or implants] knowing that it’s obvious to experience minor side effects.” – 25 years	
Help to dispel myths	
“I have felt that family planning is good. It was not like what people rumor about in the community that family planning is bad, that it has bad side effects which can even kill you. For example, like the implants, that if you are inserted wrongly you can lose your arm. I have learnt today from that tool that it is not like that.” – 20 years	
Empower women to make informed decisions	
“Because it is not time consuming, it is empowering the women, they read for themselves they have the pictures and everything.” – 18 years	

Furthermore, women described gaining insight on realistic expectations of side effects, which resulted in some women changing their minds about the method they intended to use after using iMACC. Women commented on how the information about potential menstrual changes that are common with some contraceptive methods are undesirable. They said that learning about the side effect profile of multiple methods helped them make a more informed decision about selecting an appropriate contraceptive method enhancing their self-efficacy. One woman described how iMACC led her to select an implant as her method of choice:

*“It has changed my mind because I was contemplating continuing with Depo because I had used it but I used to bleed a lot, it’s like there was something that the providers were supposed to do and they didn’t when I complained. But now after reading this I think I want to go for implants.” – Female participant, 20 years*

#### Dispelling common myths and misconceptions

All participants thought the decision aid had the potential to dismiss myths and misconceptions about FP methods and help women make informed choices after receiving information. One woman revealed one of the misconceptions she had before using iMACC, *“I did not know about the fact that IUCD, you can get it removed any time. I always thought that once you insert it then you have to go with it full time.”* Another woman described how rumors circulating in the community had discouraged her to use contraception, but the decision aid encouraged her to use FP after realizing these rumors were not true.

*“I would like to use family planning. It was not like what people rumor about in the community that family planning is bad, that it has bad side effects which can even kill you. For example, like the implants, that if you are inserted wrongly you can lose your arm. I have learnt today from the tool that it is not like that.” – Female participant, 20 years*

Providers in our study reaffirmed that there were several widespread myths that prevailed among women in the community, and thought the decision aid would be beneficial in dispelling myths. They said some women do not use IUCDs because they believe it will cause persistent backaches, prohibiting women from doing hard work. Providers also said women fear injectables may reduce libido and fear implants moving from their arm to the brain, and these fears prevent some women from using injectables and implants. They thought it was important to alleviate these fears and thought the decision aid had potential to serve this role.

#### Improvement in quality of client-provider interactions

The majority of women and providers discussed the potential utility of the decision aid in improving client-provider interactions. Overall, most providers felt the decision aid would allow women to ask more questions before they made contraceptive decisions “*because they will have the information we will be able to exchange, ask questions and interact more.”*

However, some providers disagreed, and thought the majority had already decided which method to use before they even came to clinic: *“By the time they will come to us, they will be well vast with the FP knowledge. So, they will just say I want Depo and I will have to give Depo”.*

FP providers acknowledged that it is a challenge to provide counseling on the full spectrum of FP methods when there were method stock-outs or heavy workloads, leaving little time to provide FP counseling services. Providers believed iMACC would allow them to provide systematic, comprehensive information on all contraceptive methods regardless of methods are in stock or not and without needing to rely on their memory alone, reducing the likelihood of forgetting to discuss methods that are of interest to women. One provider reflected on her experience providing FP counseling during a busy workday:

*“You see like now, in our daily practice, if there are some methods that we don’t have at that particular moment, you may forget to talk about them but this one is systematic, it’s flowing. So, whether you have it, or you don’t, that client will get that information” – FP provider, 55 years*

## Discussion

In this evaluation of a client-facing contraceptive decision aid application in Kenya, both women and providers found it to be feasible, acceptable, and useful, and had a potential to influence decision-making and reduce provider’s workload. Confidentiality was an appealing feature for both women and providers. The decision aid includes content on attributes that are important to women during the postpartum period (e.g., effect on breastfeeding, return to fertility, side effects) when making decisions to begin or resume contraception, which suggests it may be useful for postpartum contraceptive counseling.

The iMACC decision aid was developed using a combination of three theoretical frameworks previously shown to impact individual behavior change and beliefs. We did an extensive literature review of the multiple behavioral frameworks/models to identify the elements that have shown effectiveness in changing contraceptive behavior of an individual and used those elements to develop an integrated theoretical framework used in this study. Elements of all frameworks were well represented by the narratives of women in our study. Women explained how using the FP decision aid enhanced their understanding of benefits and potential side effects of contraceptive methods, and dispel contraceptive myths; this led to a feeling of empowerment to make informed decisions. We adopted the behavioral belief element from the theory of planned behavior that changes the intention towards contraceptive use. Other contraceptive studies using this theory have found that use of this theory did predict and change the intention of contraceptive use in Uganda and Ethiopia [31, 41]. The self-efficacy aspect of SCT was also included. Perceived self-efficacy of women have been shown to predict modern contraceptive use in the previous studies done in Kenya, Ethiopia and Nigeria [42, 43]. Finally, we incorporated perceived barriers to FP use from HBM into the decision aid. Other FP intervention studies using this theory have shown greater FP use among women with greater perceived benefits and lower perceived barriers [30, 42].

Our decision aid is a novel tool that could address significant gaps in FP delivery programs in LMIC settings. It provides postpartum women with a tailored and personalized contraceptive counseling experience using an interactive platform; it allows women to use it independently and select a FP method of their choice. Inclusion of providers allowed us to explore whether the decision-aid had perceived usefulness, and addressed common problems with current counseling practices. While the decision-aid is designed for postpartum women, our findings that the decision-aid was found to be acceptable by women and providers lends support for integrating this type of counseling support into routine clinical care. Various decision aids are used in the clinics in Kenya to provide FP counseling to the women. Decision aids (e.g., WHO Decision Making Tool [25], WHO Tiered Effectiveness Chart [44], Balanced Counseling Strategy [45]), commonly used in settings like Kenya include flipcharts, charts, or counseling cards. These tools are used during clinical encounters, but are primarily designed to support provider counseling, rather than client-facing tools to help women make decisions. Some other mobile interventions implemented in Kenya such as short-message service (SMS) and the mobile 4 Reproductive Health (m4RH) platform also provides essential information on contraception; however, they too are shown to increase FP knowledge but insufficient to help in making decisions [46]. While a mobile job-aid in Tanzania was an effective tool to provide FP counseling, it was designed as a tool to support community health workers (CHWs) in the delivery of services rather than helping women in making decisions [52]. iMACC may address postpartum women’s informational needs, by providing a platform for confidential counseling and helping them make a decision that meets their individual needs and values, while also complementing provider counseling. In addition, iMACC has potential to utilize the time women spend waiting to see a provider to deliver contraceptive counseling, maximizing time with providers to discuss specific questions and concerns, and saving providers time by streamlining the counseling process. Currently, providers offer individual counseling on FP, sometimes with the assistance of flip charts or charts. However, the quality of counseling is provider dependent. iMACC offers an opportunity to automate counseling and ensure key topics are uniformly discussed with all women, as well as focusing on methods and concerns women have about using specific methods.

Results from this pilot study suggest our FP decision aid is acceptable, useful, and feasible to use in resource-limited settings. To optimize the effectiveness of the tool, some refinements were suggested. The decision aid was perceived to have potential for being more useful if interactive multimedia such as audio or videos could be incorporated to overcome the barriers of illiteracy and lack of familiarity with the technology [47]. We also identified several areas where participants felt that information was missing, was not required, or was not detailed enough. Adding content that addresses lack of detail and omitting information deemed not important are expected to further enhance the acceptability of the tool.

Our study had several strengths and some limitations. We included postpartum women in different age groups and FP providers to assess acceptability and feasibility from women and provider perspectives. Participants from both rural and urban settings were included to capture perceptions from diverse settings within Kenya. We used an integrated theoretical framework to develop iMACC content that is hypothesized to guide contraceptive use among women. Our study also had few limitations. The content analysis used in this study was purely a descriptive analysis that was limited by the content available and may not completely reflect the underlying view of participants towards the FP decision-aid. Also, although our study participants were sampled from both urban and rural government-run public health facilities serving low to middle income populations, findings from four MCH clinics with a small number of women and providers may not be generalizable to other settings or groups of women.

## Conclusion

The iMACC decision aid is an innovative and informative FP tool that has potential to provide tailored contraceptive counseling to postpartum women. Our findings suggest an algorithm-based decision aid to provide contraceptive counseling may aid in appropriate selection of contraceptive methods, potentially improving continuation rates and satisfaction. Further research is needed to confirm our findings of acceptability, feasibility, and utility and evaluate the effectiveness of decision aids to support contraceptive uptake and use among postpartum women.

## Data Availability

Data are available from the corresponding author on reasonable request.

## Appendix

**Table 3:** Summarized IDI Guides

Women’s IDI topics and key interview questions	
How was your experience using FP decision aid?	
Were you able to navigate the aid and find the counseling section?	
How do you feel about the information in this aid?	
How do you feel about the usefulness of this aid in decision-making?	
How do you feel about using this FP decision aid for your FP decision-making?	
What kind of information do women find helpful when they make decisions about FP?	
FP providers IDI topics and key interview questions	
How long have you been providing family planning information or services to clients?	
Based on your experience, what influences a postpartum adolescent or adult woman when she is making a choice about family planning?	
What is your typical approach to family planning counseling?	
What questions do you typically ask women during a counseling session?	
What questions women typically ask you in a counseling session? Have you heard of and/or using any other decision making and counseling tools for providing family planning counseling services? How interested do you think adolescent girls and adult women will be in using this FP decision aid?	
Were you able to navigate the aid and find the counseling section?	
How do you feel about the information in this aid?	
How do you feel about the usefulness of this aid in decision-making? Would you use this aid for counseling if it were available in your clinic?	

FP (family planning); IDI (in-depth interview)

## Abbreviations

FP: Family planning
HBM: Health Belief Model
IDI: In-depth individual interviews
iMACC: Interactive mobile application for contraceptive choice
IUCD: Intra uterine contraceptive device
LARC: Long-acting reversible contraception
LMIC: Low- and middle-income countries
MCH: Maternal and child health
PPFP: Postpartum family planning
SCT: Social Cognitive Theory
SSA: Sub-Saharan Africa
TPB: Theory of Planned Behavior
WHO: World Health Organization
